# Optimizing Saphenous Vein Harvesting with the No-Touch Technique Using LigaSure™ and Small Incisions: A Hybrid Approach for Coronary Artery Bypass Surgery

**DOI:** 10.21470/1678-9741-2025-0001

**Published:** 2025-11-17

**Authors:** Mauricio Peña Fernandez, Juan Contreras Reyes, Juan Carlos Bahamondes, Manuel Roque Cervetti

**Affiliations:** 1 Department of Cardiovascular Surgery, Hospital Dr. Hernán Henríquez Aravena, Temuco, Chile; 2 Department of Surgery, Faculty of Medicine, Universidad de La Frontera, Temuco, Chile

**Keywords:** Optimizing, Saphenous, Veins, Harvesting, Touch.

## Abstract

Our technique described below offers a reproducible, cost-effective approach for no-touch saphenous vein harvesting that can be adopted by well-trained surgical teams. The hybrid no-touch technique, incorporating LigaSure™, small incisions, and pressurized closure, achieves excellent results with minimal major and local complications. Given the robust evidence supporting improved patency and outcomes, the no-touch approach should be considered a reliable and superior option for the second conduit in coronary artery bypass grafting procedures.

## INTRODUCTION

**Table:** 

Abbreviations, Acronyms & Symbols	
CABG	= Coronary artery bypass grafting
LITA	= Left internal thoracic artery
NT	= No-touch
RA	= Radial artery
RITA	= Right internal thoracic artery
RCTs	= Randomized controlled trials
SV	= Saphenous vein

The saphenous vein (SV) remains the most commonly used conduit for revascularization in patients undergoing coronary artery bypass grafting (CABG). However, the optimal choice of conduit for CABG remains a matter of debate. Studies have shown that vein graft occlusion rates range from 5% to 13% in one month and from 10% to 15% within the first year^[1]^.

To address the issue of vein graft occlusion, the no-touch (NT) vein harvesting technique was introduced in 1996, providing a reproducible, safe, and promising alternative. However, the "Achilles' heel" of the NT technique lies in its higher rate of local complications. In a prospective randomized trial, Meice Tian et al.^[1]^ reported a 10.3% incidence of leg complications using the NT technique compared to 4.3% with conventional harvesting.

Given these findings, our aim is to describe our particular approach to SV harvesting using the NT method with LigaSure™ and small incisions. This technique represents a hybrid option between conventional and endoscopic vein harvesting, aiming to balance safety, efficacy, and complication rates (Video 1).

**Video 1:**
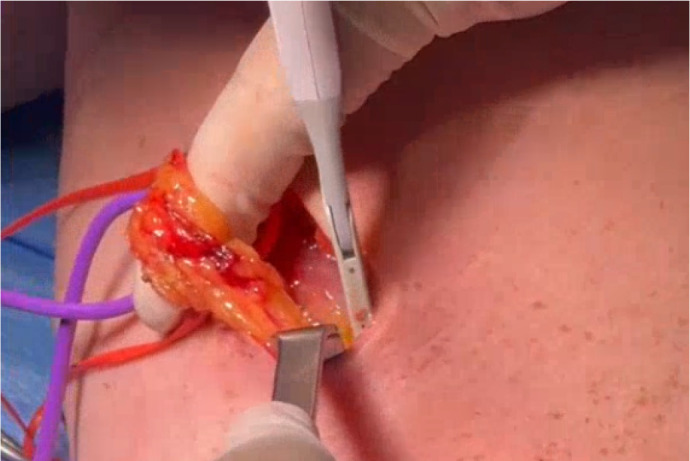
Saphenous vein graft with “no-touch” technique. Step-by-step of a hybrid technique.

Link: https://youtu.be/wLiFvZm4XTw

## TECHNIQUE

### a. Ultrasound Control:

Perform a preoperative ultrasound evaluation of both legs in the operating room to select the most appropriate SV. Selection criteria include veins with minimal collateral branches, no double echogenic halo, absence of tortuosity, and diameters ranging between 2.5 and 5 mm.

### b. Patient Positioning:

Position the patient with the leg flexed and a support placed beneath the knee to enhance stability and improve exposure of the surgical field.

### c. Initial Incision:

Initiate the procedure with the first incision located proximally, close to the groin.

Tip: Prior to incision, mark the exact location using an ultrasound-guided marker. If ultrasound is unavailable, the incision can be made two finger-widths medial to the location where the femoral pulse is palpated. The incision should be approximately 5 cm in length, ensuring that a 5 to 10 cm skin bridge is preserved to provide added strength and protection against wound dehiscence and infection.

### d. Initial Dissection:

Perform dissection using cautery until the interfacial or saphenous compartment is reached, where the SV is located.

Tip: Utilize a self-retaining retractor fixed to the skin or held with the non-dominant hand to improve visualization and provide a wide field of view, while using the dominant hand to operate the cautery.

### e. Identification of the Pedicle:

Release the venous pedicle and manipulate it gently with a vessel loop.

Tip: Perform a small dissection with cautery to identify the vein, which will provide the necessary mobility for advancement with the LigaSure™ scissors.

### f. Middle Incision:

Proceed with the middle incision, applying the same technique as described for the initial incision.

Tip: To maintain the trajectory of the vein, use a Farabeuf retractor to elevate the adipose tissue and employ blunt dissection. This maneuver ensures that the incision remains aligned and precise.

### g. Final (Lower) Incision:

Make the third incision distally, near the knee.

Tip: The NT technique is not recommended below the knee, as it increases technical difficulty. The segment between the groin and the knee is typically sufficient for performing multiple coronary bypass grafts.

### h. Closure Technique:

For wound closure, it is recommended to perform the closure before the administration of heparin or after the administration of protamine. After confirming adequate hemostasis, close the incisions with a continuous suture using 2/0 or 3/0 VICRYL®, depending on the quality and thickness of the tissue. The closure should be performed in two planes:

• Deep Plane: This plane closes the saphenous compartment with a continuous suture, incorporating part of the saphenous fascia that remains undamaged by dissection.

• Superficial Plane: This plane allows for adequate closure of the dermis. Depending on dermal thickness, 3/0 MONOCRYL®, surgical staples, or a combination of both may be used if the incision is under tension.

Tip: Before closing the final incision, it is advisable to insert an aspiration probe. The vacuum generated by the probe reduces the risk of cavity formation within the wound and lowers the likelihood of dehiscence.

### i. Postoperative Care:

Postoperative care includes the application of an intermittent compression bandage for up to three months, particularly during periods of standing. Additionally, the affected limb should be elevated to reduce the risk of edema, and it is important to keep the surgical wounds dry to prevent moisture accumulation.

## DISCUSSION

Arterial conduits are widely regarded as the gold standard for graft quality and long-term outcomes in CABG, particularly in terms of patency and survival. However, randomized controlled trials (RCTs) have struggled to conclusively demonstrate the superiority of arterial conduits over the SV. The Arterial Revascularization Trial (or ART), one of the most prominent RCTs, revealed a 14% crossover rate from bilateral internal thoracic artery to single internal thoracic artery, which has been suggested as a potential explanation for its neutral results^[2]^.

While the patency and quality of the left internal thoracic artery (LITA) are undisputed, the right internal thoracic artery (RITA) has shown more heterogeneous results. A likely explanation is that RITA harvesting is technically demanding and not "surgeon-friendly", requiring a high degree of expertise. Importantly, RITA patency has been shown to depend significantly on the surgeon’s experience and proficiency^[3,4]^.

The Radial Artery Patency and Clinical Outcomes (or RAPCO) trial demonstrated the superiority of the radial artery (RA) over the RITA as a second conduit. The estimated 10-year graft patency was 89% for the RA *vs.* 80% for the free RITA, with patient survival at 10 years reaching 90.9% in the RA group compared to 83.7% in the RITA group^[5]^. Similarly, Gaudino et al.^[6]^, in a meta-analysis evaluating the patency of second conduits, reported that only the RA and NT vein grafts were associated with significantly lower graft occlusion rates. This analysis, encompassing 14 RCTs, challenges the conventional assumption that RITA should be the natural second conduit of choice.

Given the infrequent use of more than one arterial conduit for CABG (< 10% in North America), the NT technique for SV harvesting is emerging not as a novel option, but as a compelling second graft choice. This is supported by its reproducibility, patency rates, and long-term results^[5]^.

In a landmark trial, Meice Tian et al.^[1]^ randomized 2,655 patients undergoing CABG into two groups - NT *vs.* conventional vein harvesting. At both three and 12 months, the NT group demonstrated significantly lower vein graft occlusion rates (three months: 2.8% *vs.* 4.8%; 12 months: 3.7% *vs.* 6.5%). Furthermore, recurrence of angina at 12 months was lower in the NT group (2.3% *vs.* 4.1%). However, the NT technique was associated with a higher incidence of leg wound surgical interventions at three months (10.3% *vs.* 4.3%).

Ninos Samanos et al.^[7]^ conducted a randomized study involving 156 patients divided into three groups: conventional, intermediate, and NT vein harvesting. In the conventional group, the SV was stripped and distended; in the intermediate group, it was stripped but not distended; and in the NT group, the SV was harvested intact with a surrounding fat pedicle. The patency rate in the NT group (83%) was significantly higher than in the conventional group (64%) and was comparable to the LITA (88%).

We strongly advocate for the NT technique based on its multiple benefits. This approach preserves the adventitia and endothelial integrity of the SV, thereby slowing the processes of intimal hyperplasia and atherosclerosis^[8]^. The vasa vasorum plays a crucial role in supplying oxygen and nutrients to the vessel wall, a function that is particularly relevant in the SV due to its more prolific and deeper microvascular network compared to arterial grafts. Conventional SV harvesting disrupts this network, compromising transmural blood flow and promoting neointimal hyperplasia and atheroma formation. Additionally, the preservation of nitric oxide synthetase within the endothelium and the adipose pedicle may provide further protective effects against graft failure.

## CONCLUSION

Our described technique offers a reproducible, cost-effective approach for NT SV harvesting that can be adopted by well-trained surgical teams. The hybrid NT technique, incorporating LigaSure™, small incisions, and pressurized closure, achieves excellent results with minimal major and local complications. Given the robust evidence supporting improved patency and outcomes, the NT approach should be considered a reliable and superior option for the second conduit in CABG procedures.

## Data Availability

The authors declare that data sharing is not applicable to this article as no new data were created or analyzed.

## Artificial Intelligence Usage

The authors declare that no artificial intelligence tool was used in the preparation of this article.
